# Histological Methods and Results

**Published:** 1891-11

**Authors:** L. C. Ingersoll

**Affiliations:** Keokuk, Iowa


### HISTOLOGICAL METHODS AND RESULTS.

BY L. C. INGERSOLL, KEOKUK, IOWA.

WHEN we come to the discussion of a subject about which there are conflicting opinions, it is necessary that there should be a substantial agreement as to the sources of information and the kind of testimony to be admitted in deciding the question.

If there be not this agreement, and a definite understanding arrived at as to the character of the testimony, a satisfactory decision can never be reached.

It seems to be a foregone conclusion with many scientists that, in the investigation of histological questions, the revelations of the microscope are to be accepted not only as legitimate, but as superior to all other testimony; still further, that what the microscope does not reveal is not to be accepted.

If this position be taken, it becomes evident at once that the *ultima thule* of histology is circumscribed by anatomy, and the investigations of the microscope must be confined to lifeless, dead matter, and physiology must step aside and offer no testimony in a histological case. Is it true that physiology and anatomy are so distinct from each other that function has nothing to do in determining structure, and that structure has nothing to do in determining function? It requires both structure and function to form a living organism, and the organism must be studied in full view of both, and in full recognition of their harmonious teachings.

The teachings of the microscope must be made to harmonize with those of function, and those of function must be made to harmonize with the teachings of the microscope, while to each is accorded its separate work in furnishing testimony leading to a common conclusion.

But microscopists are inclined to magnify the knowledge gained through the microscope as so superior to all other means of information as to deny the right of any one to bring testimony from any other source, should it chance to be conflicting or any departure from the testimony of the microscope.

It is no wonder that microscopists should have a sort of worshipful feeling towards the microscope with its wonderful lenses, as the great revealer of hidden mysteries. Still, it will not answer for a man to deny the conceptions of the mind gained without it, or the deductions of reason based upon established facts. There is such a thing as a mental microscope, and such a thing as the telescope of the mind. If one does not first use his mental microscope, his instrument of brass and glass will be of very little value to him. The latter instrument is but auxiliary to and corroboratory of the evidence of the former in most cases where life and function are concerned.

The microscope may reveal many things unerringly within its own peculiar realm, where it deals with inorganic matter, form, and structure. But function is out of its province; what function reveals may or may not be within its realm. And what the mental microscope and the deductions of reason reveal may be entirely out of the reach of the instrument of brass and glass for a time, then burst into view in the field with a *form* and *structure* that the mental, deriving its conception from function, had never before seen.

For example, Dr. Heitzmann claims to have discovered through this instrument a reticulum in protoplasm, a structure in "living matter,"—a definite arrangement of granules and interconnecting threads in the amœba. Frommann claims to have seen the same; and perhaps Stricker and others. Would these distinguished gentlemen of science and skill allow any one, not a microscopist, to say that he saw this structure before they did?—not *as they* did, outlined and defined, but as a necessary part of the expression of vitality.

When it was said that the earliest embryonic form of organic tissue was but a minute mass of jelly endowed with life and capable of the manifestation of life, I accepted it as determining the origin of vital tissue. But when it was further said by microscopists that this jelly-like mass of living matter is structureless, I could not accept it. Not that I had looked through the microscope with better eyes than others, but the statement did not satisfy the mind. The statement did not contain a fully rational idea.

With this conception of a formless mass of matter before me capable of the manifestation of life, my reason used the astounding fact in drawing a conclusion, thus: If this amœba, this bit of protoplasm, is capable, by an inherent power within itself, of manifesting life, it must have some method, some manner, some means, some way by which it manifests the fact that it has life.

The human mind can have no conception of a movement without some means by which the movement is produced. If the amœba changes its form, it must do it by some method of connecting together its remote parts in harmonious movement, each part with every other part. This implies, necessarily, the existence of a structure,—does not define the structure, but declares its existence.

Thus the grand fact of structure in embryonic elements is brought out by the microscope of the mind, leaving to the instrument of brass and glass the more satisfactory discovery of its form and the general outlining of its parts.

Again, we reason from function that there is a circulatory system in dentine. We do not ask the microscope to define and map it out to the eye before we believe it. We know it in advance of observation. We know by that instrument the primary fact that there is a condensation of peripheral dentine; that the tubules of the dentine are filled up late in life with lime-salts at their outermost extremities, and from this primal fact we make the logical deduction that there must be a circulatory system through which the lime-salts are carried; and we know this whether the microscope reveals it or not. Some day it may be able to outline it.

Now, using the same methods of investigation, let us turn our attention to the ultimate distribution of the fibres of the pulp.

The reason I have for making the foregoing remarks may not be apparent to all readers of this article.

Two years ago I had the honor of reading a paper before the Odontological Society of New York City, on "The Relations of the Dental Pulp to the other Tooth Tissues," being the second paper which the Society had invited me to read before them on that subject. I based my arguments upon the generally accepted teachings of histology relating to other similar tissues, on the physiological function of the tissue in question, and on clinical experience.

In the discussion of the subject, almost no notice was taken of my argument from physiological function supported by clinical experience: function was combated with the microscope, facts ignored, and logical conclusions met by dogmatical denial and assertion without demonstration or proof. Dr. Heitzmann kindly excused my ignorance on the ground of my confession that I was not a microscopist,—intimating that other sources of information and other methods of investigation are not to be admitted in the study of histological subjects, thus setting aside clinical experience and function because they do not come within the field of the microscope, and saying of himself, "I am not a dentist, and simply judge from what I have seen under the microscope."

Dr. Sudduth betook himself to the *argumentum ad hominem*, and said of me and my paper, "When it comes to his interpretation of the histology of the pulp and its ganglionic character, and his conclusions in that direction, he is treading on ground that belongs to Dr. Heitzmann and myself. He is going into the special field of the microscopists, and we must stop him, as he does not claim to be one of them." Again, after complimenting my physiological study of the pulp, Dr. Sudduth says, "Dr. Ingersoll does not claim to be a microscopist; and if he had not stepped over into the special field of histology, I should not have had anything to say regarding his views on the subject."

In January, 1890, nine months afterwards, the same subject was under discussion by the Society, introduced by a paper by Dr. George McCausey, in which Dr. McCausey, in speaking of the sources of knowledge, says, "The human animal acquires knowledge through the medium of the five organs of sense." Had he consulted mental philosophy, he would have found added, consciousness, reflection, and reason. The omission is, I think, characteristic of microscopists.

Then, again, he said, "In a consideration of the relations of the tooth-pulp to the other tooth tissues, the writer assumes that the microscope is the only medium through which we can arrive at facts;"—discarding, therefore, function and rational deductions, he turns to that alone, and impliedly rejects all declared facts unless they can be demonstrated by the microscope, and pronounces the Scotch verdict, "not proven," on everything not shown by that instrument. One word about the Scotch verdict that my microscopical friends have taken undue advantage of. It would have served their purpose better had it been written *not demonstrated*, instead of "not proven." Many facts can be proven which cannot be demonstrated. A fact is proven when sufficient evidence has been adduced to carry conviction to the mind of its truth, whether the fact can be demonstrated or not. Courts of justice recognize this, and act upon it. I may become thoroughly convinced of the fact that an insect has eyes, although I may not be able to demonstrate it by the observations of the microscope. I may become perfectly satisfied that an amœba has a digestive system, although Dr. Heitzmann cannot find any digestive apparatus with the microscope. He may deny that it has any nutrition because he cannot see any blood-vessels. I may become satisfied that the amœba has powers of locomotion, although no one should be able to see and define its *pseudopodia*. The trouble of the microscope in these cases is, that it cannot see function, nor the mental processes by which we become convinced that protoplasm exists by the nutrition of digested food, just as every other living tissue of the body exists; and we become convinced of this before the microscope has demonstrated the existence of a digestive system. The fact is proven, though not demonstrated.

So the astronomer proves the existence of a new planet before he can demonstrate it. He does it by a process of reasoning from observed facts,—facts already demonstrated. He knows well one planet in the heavens, and has studied its movements in view of others already known to be within its range of attraction. In tracing its orbit he has discovered an aberration which known forces will not account for. In studying it he brings mathematics into use, and calculates the exact extent of the aberration and the attractive force necessary to produce it. Having gained these facts by an intellectual operation, he uses his reason and arrives at the conclusion that in the direction of the aberration there must be another planet. No one has as yet seen it with the telescope, and because of this, some, who believe that the telescope is the only means of studying astronomy and not understanding the mental processes by which truth is discovered, will deny the existence of the planet because they have time and again pointed their telescopes into that part of the heavens and have never observed it. Twenty years afterwards comes the demonstration. The thoughtful and studious astronomer, who has calculated the time of the appearance, and therefore knows when and where to look, points his telescope in the right direction, and demonstrates to all observers what he had already proven by the deductions of reason.

It is very common for microscopists to assume that what appears to them in the field of the microscope is fact and certainty, and to trust to no fact concerning material existence that they cannot see and demonstrate with it,—this, too, in the face of all history since microscopical observations have been given to the world. The past is full of change of histological notions, theories, and asserted facts concerning almost every tissue of the body, and to-day histologists of equal skill in the use of the microscope differ widely as to what may be seen through the instrument, and differ widely, also, as to their interpretations of what they see.

The inerrancy of the microscope can more readily be accepted than the inerrancy of the interpretations of microscopists. Their interpretations may well be questioned when found to be not in harmony with well-known physiological function and the law of physiological development.

Since John Tomes's and Huxley's remarkable discoveries and demonstrations of the existence of a fibril within and passing through the tubules of the dentine, the profession have pretty generally assumed that if the Tomes fibre was not itself wholly nerve-fibre, it at least enveloped a nerve-fibre, although Tomes himself never ventured to affirm that he considered the fibre nerve-tissue. He did, however, say that, "When a fibre is broken across, a minute globule of transparent but dense fluid may sometimes be seen at the broken end." This is a phenomenon of the same chararacter as has often been observed when a nerve-fibre is parted.

The various phenomena which have appeared to the observation of the dental operator have been interpreted in harmony with the idea that the fibre had nerve sensation at least; and, so far as I know, no serious doubts have been entertained by clinicians concerning the existence of nerve-tissue in some form within the tubules of the dentine.

Within a few years histological students and experts with the microscope have expressed the strongest possible doubt, amounting almost to a denial, of the existence of nerve fibrils in the dentinal tubes, solely on microscopical grounds,—that is, they cannot see them. Thus, this becomes an interesting field for scientific examination.

For a brief study of the nature of the pulp processes ramifying the dentine, let us begin first at the pulp end of the fibril and observe some of its relations to the pulp, so called; then pass to the other end of the tubule, where we find a peripheral distribution of the pulp processes.

I may be allowed to say, in this connection, that in the discussion before alluded to, both Dr. Heitzmann and Dr. McCausey misrepresented me in saying that I "maintained that the whole pulp is nerve-tissue." This is the result of great carelessness, to say the least, in hearing and reading what I have said and written on the subject.

I think there is no question in the mind of any reading dentist that, however well filled the central cavity of a tooth may be with blood-vessels, myxomatous matter, connective tissue, odontoblasts, and other tissue cells, there are also nerves and nerve-fibres ramifying the mass in all directions. These have been traced from the point of entrance—the apical foramen—into the body of the organ, and in every direction to its periphera and the wall of dentine enclosing the mass usually called the dental pulp.

The question in doubt concerns the termination of these nerve-fibres. Do these nerve-ends turn backward in loops and inosculate with each other? or do they enter the odontoblast layer and become lost in the connective tissue? Thus their endings would nowhere be found. Some say that they have traced them into the individual odontoblasts. Others think that they make their appearance beside the odontoblasts and between their projecting processes.

Some assert that they can trace them through the odontoblasts and to the entrance into the tubules of the dentine, but no further. There they lose sight of them.

Gray says, "The caudate or stellate nerve-corpuscles have tail-like processes issuing from them, some of which terminate in fine, transparent fibres, which become lost among other elements of the nervous tissue."

Bödecker says, "The dentinal fibres lie between the odontoblasts," then adds, "Franz Boll saw the nerve-fibres becoming axis cylinders between the odontoblasts, and in some instances he has followed them into the dentinal canaliculi." "Although I can furnish no direct proof," says Bödecker, "yet I regard the prolongation of the nerve-fibres into the dentinal canaliculi as certain."

Klein, in the "Atlas of Histology," says, "The assertion of Boll may be correct, that the non-medullated nerve-fibres ascend into the dentinal canals."

J. L. Williams says, "My studies of the pulp-tissue have led to the discovery that the odontoblasts are probably of the nature of multipolar ganglion cells. The odontoblasts are composed of what is essentially neural matter."

Boll writes, "That at least one of the processes of a multipolar nerve-cell does not branch, but becomes continuous with a nerve-fibre."

Dr. McCausey says, "The odontoblasts are protoplasmic bodies; that their processes are continuations of the body, and may be traced into the cap of dentine,"—does not believe that the processes are in any part nerve-tissue. Then observes, "If the pulp be a nerve, we shall expect to find it composed wholly of the elements which enter into the structure of nerve-tissue;" thus setting up a man of straw that he could easily tumble down, for neither I, at whom he aimed, nor any one else, ever took the position which he assumes,—that the pulp is *wholly* nerve-tissue, and that, therefore, the processes must be. All that is desired in this connection he fully maintains, and Dr. Heitzmann the same, that "The pulp, like other tissue, contains neural tissue of that form which enters into the structure of nerves whose function is the conduction of nervous influence,—medullated nerve-tissue."

Now we will again bring microscopical observations to our aid in learning the nature of the tissue at the other end of the dentinal tubes, which I and most others call the peripheral end of the dentine fibrils, although Dr. Heitzmann insists that the pulp itself is a peripheral organ.

This brings us into the region of the interglobular spaces of Czermak and their soft tissue contents. Here, too, we find a variety of opinions. Czermak himself scarcely ventured an opinion as to the contents of the spaces. John Tomes and Perpinge called it the "granular layer." Charles Tomes called it "transitional tissue," and again, finding bay-like excavations filled with soft tissue, he called it "protoplasmic cell-tissue."

Bödecker said that what Tomes saw was "basis substance of the dentine devoid of lime-salts."

John Tomes says that protoplasmic matter is enclosed within the loops of the dentinal fibrillæ; that he has seen the fibres pass entirely through the spaces and out into the dentine.

R. R. Andrews speaks of "large nucleated bodies in the cementum, larger than the odontoblasts, whose function is the forming of the cement cells, having processes, some of which run in the direction of the termination of the dentinal tubes, as though connected with them."

Wedl called the soft substance in the interglobular spaces "amorphous substance."

Let us sum up now what we have found at the extremities of this delicate fibre, the so-called dentinal fibril. We find at one extremity, in the body of the pulp, mucoid substance, protoplasm, myxomatous matter, nerves, nerve-threads and fibres in great number, connective tissue and connective-tissue cells, multipolar cells, vesicular nuclei in the peripheral cells, and neural tissue of that form which enters into the structure of nerves whose function is the conduction of nervous influence. We have then, in the pulp, all the functional organism and all the material needed for the construction of nerve-tissue.

At the other extremity, according to the histologists named, we find transitional tissue, protoplasmic tissue, embryonal tissue, basis substance of dentine, nucleated bodies, and amorphous substance.

Here also at the peripheral extremity we find the same embryonic material, the same unorganized material as at the pulp end; so that if there is not nerve-tissue in the dentinal tubules, it will not be for want of functionating power or embryonic material at hand to be metamorphosed into nerve-tissue.

Now let us leave for a while the microscopical aspects of the case and turn our attention to the demands of physiology,—not decide the case on the testimony of microscopy alone.

Physiology teaches us that every organ of the body, great and small, every functionating part is brought into the exercise of its function by the nervous system acting as an excitant; that for this purpose minute threads and fibres of nerve ramify every tissue of the body, and cause all the functional activity of all the organs; that harmony of action is absolutely essential to completeness of the organism as a whole. When, therefore, we are told that there is any part of the organism not penetrated by nerve-fibre, and yet performs a function, physiology responds in the negative.

We find that the odontoblasts on the pulp-periphera are active workers, are builders of the dentine. According to physiology, they perform this function under the stimulus of nerve influence, which acts by presence and contact. We find that these dentine-building organs have processes which are developed as the work progresses. We find, also, that the functional work of the odontoblasts is carried on with equal facility at the extremities of their processes and at their bases. From a physiological stand-point, therefore, the only rational conclusion we can reach is, that there is nerve stimulus at the terminal ends of these processes, be they long or short; and if so, the fibre of nerve must have travelled the length of the dentinal tube during the dentine-forming stages to reach that terminal end to stimulate its functional work. That functional work of the pulp processes is being performed all along the line up to and beyond the mature age of the individual.

Let us now learn what physiology has to offer to supply this demand for nerve influence through such fine working threads of tissue as the pulp processes.

I wish, first, to call attention to a few axiomatic facts. All the physical force of the body, all the manifestation of functional activity, is generated in and applied by the nervous system.

For the performance of all the varied functions of the human body, the nervous system offers but one kind of tissue. That is to say, there is no difference in the nerve-tissue of the different classes or pairs of nerves that proceed out from the brain, whatever the organ they reach or whatever the function they stimulate. Nerve-tissue exists, however, in different forms, such as vesicular, tubular, and fibrous, held in place by the everywhere-present connective tissue, with possible exceptions,—the amount of connective tissue having its maximum and minimum according to the size and strength of nerve-tissue demanded by the function.

For physiological study, a convenient division of the nervous system is made into *ganglia*, *nerves*, and *nerve-ends*.

Too little has been said of the nerve-ends, probably because so little is known of the nerve-endings. It will not answer, however, to say that they end nowhere,—that their endings are *lost* in the tissues. Microscopically it may answer to say that they run out into nothingness and are lost, but not physiologically. They may be lost to the sight of the microscope, but not lost to physiological function. It is the nerve-ends that take the impressions of sight, sound, and feeling.

It is well known that nerve-filaments do not branch except at their endings. Bundles of fibres may branch, and the individual fibres become brush-like at their endings, where the force generated in the ganglia is expended in producing functional action.

The peripheral regions of the body and of the various organs of the body are literally filled with nerve-ends in the form of plexuses and minute ganglia. Now let me ask, Do not the filamentous plexuses under the enamel, formed by looping and decussation, seem and act, physiologically, altogether like nerve-endings?

Nerve-fibre has an ending somewhere, and that, too, as a very important part of the nerve system, a depot of nerve influence. Every railroad track has an ending somewhere, and at that ending there is a station, where are performed the various functions of the road. Microscopists take us into the grand union depot of the pulp, from which there are roads leading out in all directions; take us out through the odontoblast-yard; take us through the gate into the tunnel of the tubuli, then say, "Here the track ends, with no station in sight; you must get through the tunnel to the depot at the other end, in the interglobular spaces, by other means than a railroad track of nerve-tissue." The nerve-fibre apparently ends in the darkness, yet with no suitable termination, such as we have a right to expect.

We have now to meet the microscopical objection that this connecting link between the central and peripheral depots of nerve influence is not nerve-tissue, or not known to be nerve-tissue, although all the functions of nerve influence are carried on at each end, with evident communication between the two.

I made this issue before the Odontological Society of New York City that, if this teaching of physiology that there is a nerve connection between the peripheral region of the dentine and the central organ of the tooth be not accepted, it is incumbent upon those who do not accept the teaching to name and demonstrate the existence of some other tissue that cannot only simulate nerve effects, but actually perform the functions of nerve-tissue.

Dr. Heitzmann accepted the issue and named *living matter.* I considered this a complete evasion of the question. Of course, it is living matter. There is living matter everywhere in a living organism. Wherever there is life and growth there must be living matter. Then Dr. Heitzmann makes use of the most contradictory statements, like these: "The dentinal fibrillæ are not nerves." "Both nerves and muscle are living matter." "Nobody can tell the difference between pure living matter and nerve-tissue." If that is true, by what stultification of his understanding can he affirm that the living matter of the fibrillæ is not in some part nerve-tissue?

Dr. Sudduth, also, accepted the issue and named protoplasm, and gave his reasons for supposing that protoplasm might in the economy of nature be substituted for nerve-fibre. He had experimented with protoplasm and the amœba, and found that when touched with the point of a needle they would contract,—would show signs of conducting sensation. Dr. Allan and others have considered this ample evidence that protoplasmic threads might account for the conduction of sensation through the dentinal tubes to the pulp.

Dalton tells us that muscle-fibre will contract in the same manner, and further says, "This contraction will take place under the microscope when the fibre is entirely isolated and removed from contact with any other tissue; showing that the properties of contraction and irritability reside in the fibre itself, and are not communicated to it by other parts."

The sensitive plant will contract in like manner. Touch one of its leaflets and all the leaflets on the same petiole will fold together, and the petiole itself drop downward. Does this prove or play the part of even the poorest kind of evidence that muscular or vegetable fibre might be substituted for nerve-fibre for the performance of the function of physiological sensation?

The mistake in these cases is in confounding irritability and contractility with sensation. The only evidence of irritability of organic tissue is the perceived motion of the fibre. In the conduction of physiological sensation there is no perceptible motion, nor is there any evidence that motion bears any part in explaining the philosophy of conduction of sensation. Sensation can only be manifested when and where there is unrestrained connection with the sensorium. Irritability may be manifested wholly disconnected from the sensorium.

These facts point to the conclusion that there is no other tissue than nerve-tissue that has the property of conducting physiological sensation.

The question now very naturally and fairly arises, Is it not possible that microscopists are mistaken in their interpretation of what they see within the tubules of dentine, calling it protoplasm, living matter, mucoid tissue, jelly-like substance? in each case indicating that it is a structureless, unorganized mass of embryonic matter.

To call it protoplasm is to exalt mere embryonic tissue to the performance of the function of the most highly organized of the tissues of the human body.

Let us see now if we are not offered a better and a more rational interpretation which will harmonize the teachings of microscopy with known function. Physiology teaches that the only essential part of nerve-tissue is the axis-cylinder,—that the embryonic existence of the nerve is limited to this kind of tissue, and its terminal ends are limited in the same manner.

The loss of the white substance of Schwann and of all other coatings that surround the axis-cylinder, as the nerves approach their termini, has a fit illustration in the development of the vascular system.

In examining the course of an artery from its source to its peripheral endings in capillary vessels, you lose sight, one by one, of its four distinct coatings, the *tunica*, *elastic coat*, *muscular coat*, *fenestrated coat*, and find nothing remaining for the capillaries but the endothelial layer of cells simply "glued together by their protoplasm," as Stricker expresses it. Under the microscope, therefore, arterial tissue will present a different structure at different distances from the heart.

The same will be true of the nerve-tissue in various localities. The connective-tissue coatings, existing for mechanical purposes chiefly, to hold together the delicate functionating tissue in parts where there is strain and danger of harm from accident, are not to be found in the tubules of the dentine, the walls of the tubes affording ample support and protection to the gelatinous fibre passing through the tube. With this loss of external tissue the internal fibre would appear like another kind of tissue, and take a very different staining.

Now let us look into the nature of this axis-cylinder. Dunglison says that "the white substance and the tubular sheath usually disappear as the nerve approaches its terminal distribution." He also observes, "Neurine consists chiefly of albumen and a peculiar fat." "The experiments of Brown-Sequard show that this form of nerve-matter conveys sensitive impressions."

Cleland says, "The axis-cylinder is of albuminoid composition. What is most important for a student to understand is, that every nerve in the body has an albuminoid thread, while only some are enveloped in the substance yielding protagon."

Turner, of the University of Edinburgh, says, "Although, from the great delicacy of nerve-fibre, the axis-cylinder cannot be seen in the living fibre of a cerebro-spinal nerve, there are many reasons for regarding it as a structure existing in the living nerve, and not the product of a post-mortem change."

On the above testimony of high authority we learn that the only essential part of nerve-tissue is the axis-cylinder; that the axis-cylinder is the only part of the nerve that persists in the ultimate distribution of the nerve-fibres; that the axis-cylinder is a gelatinous albuminoid nerve-fibre often drawn out to extreme tenuity; that its glistening appearance renders it so strikingly like that of protoplasm and living matter that, on the authoritative opinion of Dr. Heitzmann, no man can tell the difference between pure living matter and nerve-tissue; therefore there is not only a possibility, but a strong probability, that microscopists are mistaken in their interpretation of the contents of the dentinal tubes. And inasmuch as no other tissue than nerve-tissue has been demonstrated to possess the function of conducting physiological sensation, and inasmuch as physiological function and clinical experience unite in declaring that the dentinal fibre behaves under all circumstances like nerve-tissue, there seems very little room to doubt the existence of nerve-tissue within the tubules of dentine.

